# High-dose vitamin D supplementation is related to an improvement in serum alkaline phosphatase in COVID-19 patients; a randomized double-blinded clinical trial

**DOI:** 10.1186/s41043-023-00409-y

**Published:** 2023-07-25

**Authors:** Reza Rezvani Moghaddam, Zahra Khorasanchi, Ayad Rasool Noor, MohammadReza Shadmand Foumani Moghadam, Ali Jafarzadeh Esfahani, Abdullah Khalaf Merhej Alyakobi, MohammedHadi Lafta Alboresha, Payam Sharifan, Ali Bahari, Reza Rezvani, Malihe Aghasizade, Maryam Heshmati, Reza Assaran Darban, Gordon Ferns, Majid Ghayour Mobarhan

**Affiliations:** 1 Department of Nutrition, School of Medicine, Mashhad University of Medical Sciences, Mashhad, Iran; 2 Student Research Committee, Faculty of Medicine, Mashhad University of Medical Sciences, Mashhad, Iran; 3 Department of Biology, Faculty of Sciences, Mashhad Branch, Islamic Azad University, Mashhad, Iran; 4 Nutrition Sciences, Varastegan Institute for Medical Sciences, Mashhad, Iran; 5 Metabolic Syndrome Research Center, Mashhad University of Medical Sciences, Mashhad, Iran; 6 Department of Gastroenterology and Hepatology, Faculty of Medicine, Mashhad University of Medical Sciences, Mashhad, Iran; 7 International UNESCO Center for Health Related Basic Sciences and Human Nutrition, Department of Nutrition, Faculty of Medicine, Mashhad University of Medical Sciences, Mashhad, Iran; 8 Department of Clinical Care Medicine, Mashhad University of Medical Sciences, Mashhad, Iran; 9 Brighton and Sussex Medical School, Division of Medical Education, Brighton, UK

**Keywords:** Vitamin D, COVID-19, Liver function

## Abstract

**Background:**

The benefits and harms of vitamin D supplementation in the treatment of COVID-19 have not yet been fully documented. In this study, we aimed to evaluate the effects of high-dose vitamin D supplementation on liver function tests in COVID-19.

**Method:**

This double-blinded randomized clinical trial was conducted on 140 hospitalized patients aged > 30 years. Patients were randomly allocated to receive either intervention group (*n* = 70 receiving 50,000 IU of vitamin D capsules orally as a single dose and then 10,000 IU syrup daily from the second day of admission for 30 days) and the control group (*n* = 70 receiving 1000 IU vitamin D syrup orally per day). Liver function tests (LFT), including alanine aminotransferase (ALT), aspartate aminotransferase (AST), alkaline phosphatase (ALP), gamma-glutamyl transferase (GGT), and Lactate Dehydrogenase (LDH) were evaluated at baseline and at the end of the intervention. Decision tree analysis was performed to identify the predictors for change in liver enzymes.

**Results:**

Among COVID-19 patients, a significant decrease was observed in serum level of ALP between intervention and placebo groups (*p* = 0.04). In addition, decision tree analysis revealed that GGT, temperature, serum magnesium level at baseline and gender were the most important predictors of ALT changes in COVID-19 patients.

**Conclusion:**

High-dose vitamin D supplementation improved ALP markers among COVID-19 patients. More randomized controlled trials with longer follow-up times will be required.

**Supplementary Information:**

The online version contains supplementary material available at 10.1186/s41043-023-00409-y.

## Introduction

Coronavirus disease 2019 (COVID-19) is a rapidly spreading respiratory illness caused by the severe acute respiratory syndrome (SARS) coronavirus 2 (SARS-CoV-2) virus that has constituted a global health crisis [1]. COVID-19 symptoms spectrum is wide and can vary from no symptoms to mild, acute, and severe respiratory distress syndrome (ARDS) [2–4]. ARDS is characterized by a cytokine storm, in patients with severe disease [5], which may lead to multiple organ damage, even in cases with initially mild symptoms [2, 4]. Based on the clinical experiences during COVID-19, researchers focus their efforts on more effective treatments, identification and prevention of factors that in some cases enhance the severity of COVID-19 [2, 4, 5].

The role of vitamin D supplementation as a treatment for COVID-19 has been a subject of considerable debate [6]. Currently, determining the benefits and harms of vitamin D supplementation as a treatment of COVID-19 is in its exploring phase. It has been found that vitamin D deficiency is related to higher risks for SARS-CoV-2 infection [6, 7]. Over the last few months, some studies have hypothesized the possible beneficial effect of vitamin D supplementation in patients with COVID-19 in order to improve the immune balance and prevent the hyperinflammatory cytokine storm [8]. Among hospitalized patients with severe COVID-19, vitamin D3 supplementation was safe, but increased 25-hydroxyvitamin D levels but did not reduce hospital length of stay or any other relevant outcomes compared to placebo [9–11]. Additionally, vitamin D supplementation does not confer therapeutic benefits among hospitalized patients with severe COVID-19 [11]. Nevertheless, many authors have investigated the effects of vitamin D on infection risk reduction, however, as previously mentioned effects of vitamin D therapy in the COVID-19 era is still controversial.

With time, the understanding of the disease has improved, and it has become apparent that COVID-19 involves the pulmonary and gastrointestinal systems, the heart, and the liver [12]. Researchers have observed a significantly elevated aspartate aminotransferase (AST), alanine aminotransferase (ALT), Prothrombin time (PT), and Total Bilirubin in severe COVID-19 patients compared with patients with mild disease, and serum levels of aminotransferase are associated with both the severity and mortality of COVID-19 [12]. On the other hand, low levels of serum vitamin D were found to be associated with increased risk of elevated level of ALT, AST, or GGT [13]. However, there is insufficient evidence on the effect of vitamin D supplementation on hepatic diseases, especially among clinical trials [14]. Nevertheless, an observational report suggested that individuals with lower serum vitamin D levels have an increased risk of elevated ALT, AST, GGT, and ALT–AST, while AST/ALT ratio > 1 was associated with severe outcomes and increased mortality in COVID-19 patients [15].

Based on the evidence, the primary aim of this study was to determine the effect of oral administration of high and low-dose vitamin D on liver function tests in hospitalized COVID-19 patients. The secondary aim of this study was to identify the predictors for change in liver enzymes in hospitalized COVID-19 patients.

## Method

### Subjects

This study was conducted on 140 patients aged > 30 years who were referred to the COVID- 19 dedicated ward of Imam Reza hospital in Mashhad city, Iran, from September to December 2020. The definitive diagnosis of COVID- 19 disease in these patients were based on two criteria:

1. Laboratory results including positive CRP test and a reduction in lymphocyte count (lymphocyte > 1100/Ml) and Reverse transcription polymerase chain reaction (RT-PCR) if available.
2. Radiological findings including lung patchy infiltrations in chest X-ray or CT scan [16].

The exclusion criteria are considered as pregnancy, lactation, history of calcium lithiasis, renal failure, Cancer, known hypervitaminosis D or hypercalcemia, contraindications for consumption of vitamin D supplements, including sarcoidosis, active granulomatosis, tuberculosis, lymphoma, enrolment in another clinical trial simultaneously.

### Study design

This study was a double- blinded randomized clinical trial. Eligible patients determined at the screening visit. At baseline visit, patients who were qualified for participation in the study were stratified according to severity of disease at screening. Participants allocated based on their severity of disease (according WHO criteria [17]) into intervention or control groups based on random sampling using a random number table.

1. 70 Patients taking 50,000 IU of vitamin D capsules (ZAHRAVI, Iran) orally at the first day of inclusion as single dose and then 10,000 IU/ml syrup daily from second of admission day for 30 days (intervention group).
2. 70 Patients taking gelatin soft gel as placebo, along with 1000 IU vitamin D syrup orally per day till 30 days (control group).

Oral vitamin D supplements were given with lunch, two hours apart from other medications (if applicable). Both preparations of vitamin D were similar in taste, colour, labelling and identical manufacture department (Department of Pharmacology, Mashhad University of Medical Sciences, Mashhad, Iran). The protocol of this study has been published elsewhere [18].

**Fig. 1:**
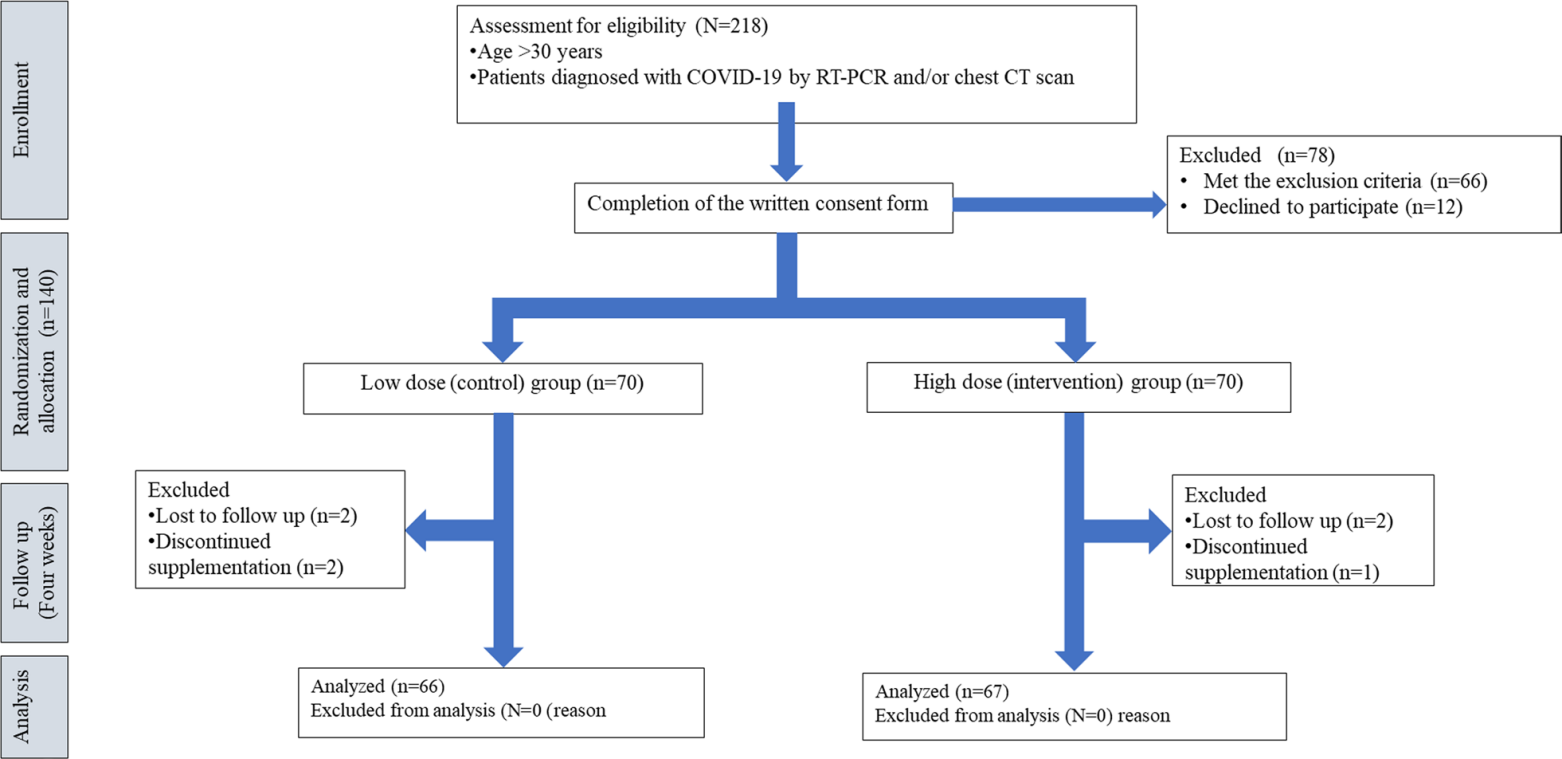
The flowchart of the study population

Figure 1 demonstrates the flowchart of participants. The CONSORT checklist was used for the present randomized trial [19].

This study was approved by the ethics committee of Mashhad university of medical Sciences (ethical code: IR.MUMS.REC.1399.237) and was registered in the Iranian Registry of Clinical Trials website (IRCT20110726007117N11). According to the beneficial effects of vitamin D in controlling the infection and the medical ethics, both groups received vitamin D supplements. Based on the Helsinki ethical consideration, all the patients signed the informed consent form before entering the study.

### Side effects

Even though no particular side effect has been reported for vitamin D supplementation at 50,000 and 10,000 IU/day, patients were asked about any side effects during follow up and no serious side effect was reported.

### Laboratory assessment

All blood samples were taken in the morning and after 12 h of midnight fasting by taking 10 mL of intravenous blood, stored in two tubes, including either EDTA for complete blood count (CBC) test or a gel tube for biochemical and hormonal tests. Liver function biochemical measurements including alanine aminotransferase (ALT), aspartate aminotransferase (AST), alkaline phosphatase (ALP), and gamma-glutamyl transferase (GGT), and Lactate Dehydrogenase (LDH), were measured using Pars Azmun test kits in BT-3000 auto-analyzer. Additionally, Serum 25(OH)D concentrations were measured using commercial ELISA kits (Pishgaman Sanjesh- Iran), using an Awareness/Stat Fax 2100 analyzer.

### Classification and regression trees (CART)

In order to determine the variables that affected the change in serum levels of liver function tests, as indicators of treatment response, classification and regression trees were performed using the rpart function of the rpart package in R version 4.1.2 (R Core Team. 2020). As the target value in the training data was categorical in this study (reduced, no change, increased), the classification was used. Classification tree uses a tree structure is used to divide independent variables into homogeneous subgroups according to the dependent variable in a hierarchical order. The dependent variables with the best separation are shown at the intermediate nodes in the tree with the critical value for the differential variables presented in the branches of these nodes. The lines from the root node (first node) to the leaves (last node) are presented along with the rules of separation.

### Statistical analysis

Data analysis was performed using the statistical package for social sciences (SPSS) software version 16 for windows (SPSS, Chicago, IL, USA). The Kolmogorov–Smirnov test was used to evaluate the normality of continuous variables. Mean, and standard deviation were used for normally distributed continuous variables, and median and interquartile range (IQR) were used for non-normally distributed continuous variables. Frequency and percentage were used for categorical variables. The linear mixed model was used to assess the effect of time, group and time-group interaction, adjusting for possible confounders. Time and group were used in the fixed-effect model, while other related variables were treated as random effect models. The best model was selected based on Akaike Information Criterion (AIC). The findings of the linear mixed model were presented using adjusted mean difference for each group from baseline to follow up and the difference between groups at baseline and at follow-up time using the Bonferroni correction as well as p value.

## Result

A total of 133 patients (75 (56.3%) male and 58 (43.7%) female) with the mean age of 59.19 ± 13.37 years entered the study. Demographic and clinical characteristics of the patients are presented in Table 1. Among the patients, diabetes was the most common comorbidity (43.4%) followed by hypertension (42.2%) and CVD (21.4%). The mean and SD for age in the intervention and control group were 58.83 ± 13.56 and 59.56 ± 13.27 years old, respectively (*p* = 0.677) (Table 1).

**Table 1:** Description and comparison of demographic and clinical characteristics of the patients between intervention and control groups

Variable	Intervention group Frequency (%) n = 67	Control group Frequency (%) n = 66	p-value
Demographic characteristics			
Age (year)	58.83 ± 13.56	59.56 ± 13.27	0.677
Gender			
Male	36 (49.5%)	39 (50.5%)	0.567†
Female	31 (53.8%)	27 (46.2%)	
Past medical history			
Diabetes	36 (48.0%)	39 (52.0%)	0.549†
Hypertension	38 (52.1%)	35 (47.9%)	0.740†
Cardiovascular disease	18 (48.6%)	19 (51.4%)	0.790†
Smoking			
Cigarette	8 (50.0%)	8 (50.0%)	0.961†
Hookah	6 (50.0%)	6 (50.0%)	0.966†
Clinical parameters and Arterial Blood Gas			
O2 Sat %	86.79 ± 7.62	86.26 ± 6.64	0.352‡
PaO2 mmHg	49.05 ± 37.49	42.63 ± 17.33	0.044‡
PCO2 mmHg	46.67 ± 39.77	40.17 ± 10.16	0.039‡
HCO3 mEq/L	27.0 ± 9.54	24.37 ± 5.45	0.002‡
Ph	7.40 ± 0.08	7.39 ± 0.07	0.547‡
Temperature	37.12 ± 0.56	36.83 ± 2.07	0.081‡
Calcium (mg/dL)	8.57 ± 0.77	8.47 ± 0.82	0.269‡
Phosphorus (mg/dL)	4.02 ± 0.95	3.98 ± 1.09	0.755‡
Magnesium (mg/dL)	2.28 ± 0.27	2.25 ± 0.34	0.459‡
Liver function tests and vitamin D			
Vitamin D (ng/mL)			
Baseline	23.34 ± 18.47	23.07 ± 16.12	0.680‡
After 1-month	34.49 ± 17.51	28.17 ± 18.60	0.008‡
ALT (U/L)			
Baseline	36.0 (21.0–68.0)	35.0 (23.0–71.0)	0.569‡
After 1-month	33.0 (16.0–43.0)	29.0 (18.0–46.0)	0.757‡
AST (U/L L)			
Baseline	40.0 (26.7–63.5)	37.0 (25.0–60.0)	0.679‡
After 1-month	21.0 (16.0–28.0)	21.0 (15.2–29.0)	0.913‡
ALP (U/L)			
Baseline	192.0 (158.0–277.5)	182.5 (136.2–267.5)	0.264‡
After 1-month	207.0 (172.5–249.7)	219.5 (174.7–270.7)	0.273‡
GGT (IU/L)			
Baseline	48.0 (29.0–87.5)	45.5 (26.2–77.5)	0.401‡
After 1-month	12.0 (8.0–19.0)	18.0 (10.0–33.0)	0.010‡
LDH (units/L)			
Baseline	432.0 (350.0–622.0)	498.5 (359.2–632.7)	0.379‡
After 1-month	359.0 (278.0–514.0)	340.0 (303.0–429.0)	0.833‡

^†^The chi square test was used for the comparison

^‡^Independent sample t-test or mann–whitney test was used for the comparison

ALT: Alanine Aminotransferase, AST: Aspartate Aminotransferase, GGT: Gamma-Glutamyl Transpeptidase, ALP: Alkaline Phosphatase, LDH: Lactate dehydrogenase

Vitamin D supplementation in the intervention group significantly increased median (IQR) for serum vitamin D from 19.82 (20.44) to 31.50 (15.62) (*p* < 0.001), while median (IQR) for serum vitamin D level in the control group changed from 21.04 (20.55) to 22.55 (18.36), which was not statistically significant (*p* = 0.340).

**Fig. 2:**
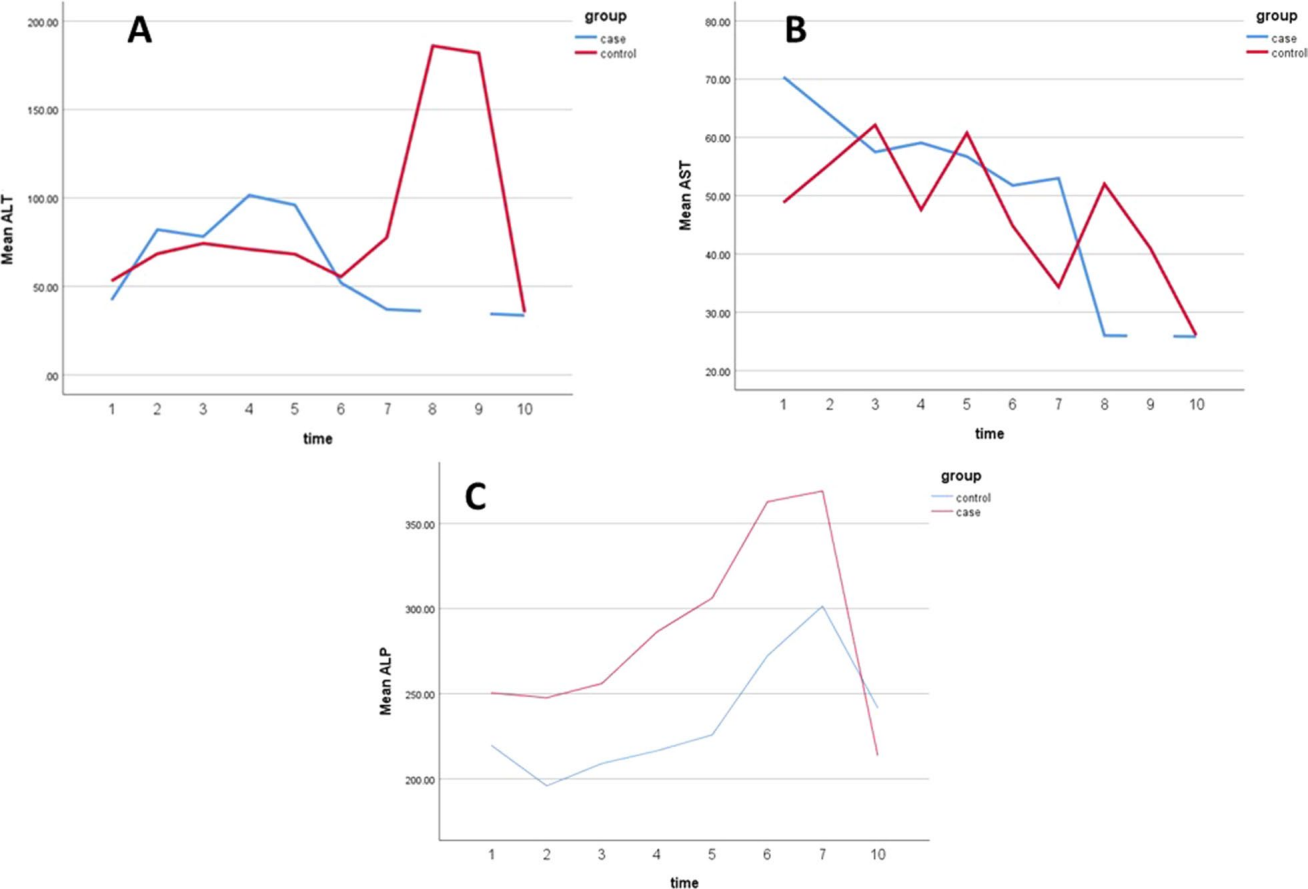
Longitudinal follow-up of ALT, AST, and ALP parameters in case and control group

Ten in hospital records the three main variables of the study were retrieved from health information system (Fig. 2). According to the longitudinal diagram 2-A, mean ALT in the intervention group had less changes and decreased more at the end of the study. Figure 2-B shows that at the beginning of the study, man AST level was higher in the intervention group, but decreased over time and reached a lower level compared to the control group at the end of the study. No significant difference was present in terms of change from baseline, and the mean baseline and 1-month values of AST, ALT and ALP. The mixed model analysis showed that time effect was significant for all study variables except for ALT (Table 2). This indicates that all LFT parameters significantly changed over time except ALT (*p* < 0.05). A significant time*group effects were present for ALP (*p* = 0.04) indicating a significant difference in changes of mean serum ALP between intervention and control groups. Also, Table 2 shows that the mean serum ALP level decreased by 6.02 units in the intervention group during 1 month compared to the control group.

**Table 2:** Mixed model analysis for longitudinal follow-up of liver function parameters

Variable		Time effect	Group effect	Time* group effect
ALT	Beta	− 1.25	10.97	− 0.32
	P-value	0.47	0.14	0.82
AST	Beta	− 2.21	1.79	− 0.22
	P-value	**0.006**	0.76	0.84
ALP	Beta	3.4	34.92	− 6.02
	P-value	0.11	0.6	**0.04**
GGT	Beta	− 1.19	− 2.71	− 0.11
	P-value	**0.004**	0.73	0.73
LDH	Beta	− 1.69	2.86	− 3.46
	P-value	**0.003**	0.51	0.67

Bold values indicate statistically significant (*P* < 0.05)

ALT: Alanine Aminotransferase, AST: Aspartate Aminotransferase, GGT: Gamma-Glutamyl Transpeptidase, ALP: Alkaline Phosphatase, LDH: Lactate dehydrogenase

**Fig. 3:**
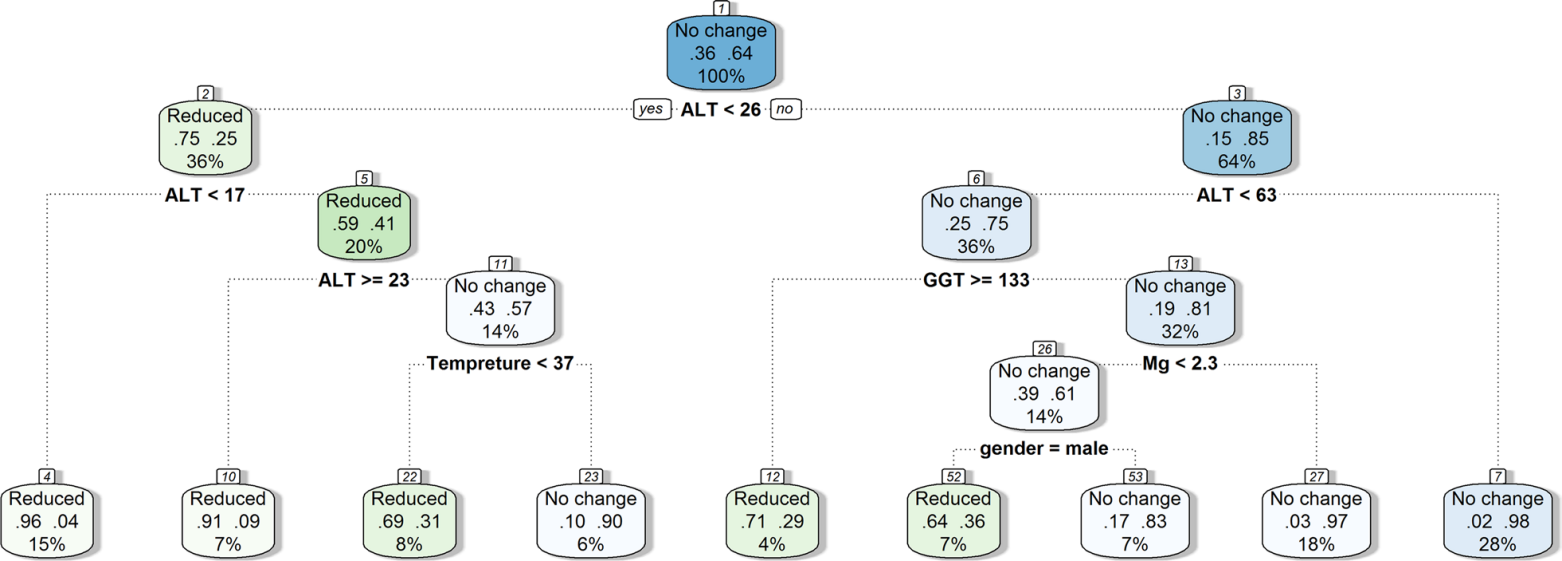
CART output illustrates a five-level decision tree. The cases partition is based on ALT, GGT, temperature, serum magnesium, and gender as prognostic factors related to changes in ALT. The hierarchy consisted of ALT at level 1 and 2, GGT at level 2, temperature and serum magnesium at level 3, and gender at level 4

Decision tree analysis was performed to identify the predictors for change in liver enzymes. In terms of ALT, the decision tree indicated that no change in ALT was predictable in patients with baseline ALT levels above 63 U/L, while ALT reduced after treatment in patients with baseline ALT levels between 26 and 63 IU/L who had GGT levels more than or equal to 133 IU/L. In patients with baseline ALT level between 26 and 63 U/L and GGT lower than 133 IU/l, serum magnesium level above 2.3 mg/dL was associated with no change in ALT after treatment, while in patients with serum magnesium levels less than 2.3 mg/dL, male gender was associated with reduced ALT after treatment (Fig. 3). In terms of AST, decision tree indicated that in patients with baseline AST levels below 25 IU/L, GGT greater than or equal to 25 IU/L, serum AST decreased after treatment, while AST increased after treatment in patients with GGT levels below 25 U/L. AST increased in patients with baseline AST levels greater than 25 IU/L and PLT greater than or equal to 112 $\times$ 103/mL. In patients with ALT greater than 25 IU/L and PLT lower than 112 $\times$ 103/mL, AST decreased after treatment in patients with serum phosphorus greater than or equal to 3.6 mg/dL, while AST increased in patients with serum phosphorus levels below 3.5 mg/dL (Additional file 1). In terms of ALP, decision tree indicated no change in ALP after treatment in patients with baseline ALP levels below 257 IU/L and serum GGT greater than or equal to 47 IU/L, while ALP reduced in patients with GGT levels lower than 47 IU/L. IN patients with baseline ALP levels lower than 257 IU/L, ALP reduced in patients with baseline PH below 7.4, and patients with PH greater than 7.4 who were mechanically ventilated. In non-ventilated patients, ALP reduced in patients with PDW lower than 12. In patients with PDW greater than or equal to 12, ALP reduced in those who had PH between 7.4 and 7.5 and in patients with PH greater than 7.5 with neutrophil count greater than or equal to 88 $\times$ 103/mL, while ALP did not change in patients with neutrophil count lower than 88 $\times$ 103/mL (Additional file 1).

## Discussion

The current study was a parallel total-blind RCT evaluating the effects of high dose vitamin D supplementation on liver function in confirmed cases of COVID-19. Our findings showed that 50000 IU vitamin D as a loading dose and followed by 30 days supplementation with 10,000 IU vitamin D resulted in a significant reduction in ALP among COVID-19 patients.

Several studies suggested vitamin D reduces the risk of COVID-19 infection, severity and mortality as the findings of other studies [20, 21]. In other words, people who have sufficient vitamin D levels are less likely to be infected and suffer a critical illness or die from the COVID-19 infection [20, 21]. There is also a possibility that vitamin D supplementation might reduce the impact of COVID-19, especially in patients and populations with a high prevalence of vitamin D deficiency [20–22]. However, some other studies have not reported a strong therapeutic benefits of vitamin-D among hospitalized patients with severe COVID-19 [11]. One of the side factors of COVID-19 infection is an abnormality of measures of liver function. Results of study showed that, severe COVID-19 has a significantly elevated AST, ALT, PT, and total bilirubin compared with mild COVID-19 patients [12, 15]. Based on this, it is essential to recognize patients with elevated liver chemistries who may experience the severe condition, and consequently, are more likely to be admitted to ICU [12]. In addition, AST/ALT ratio > 1 was linked to the more severe clinical condition and mortality in COVID-19 patients [15].

There is some evidence that high dose vitamin D supplementation could improves ALT in patients with elevated LFTs [23]. In addition, the relation between low levels of serum 25 (OH)D and elevated serum transaminases was revealed in several studies previously [24, 25]. Nevertheless, Almehmadi et al. did not find any association between Vitamin-D and ALT in COVID-19 patients [26]. There was no association between vitamin-D and AST and ALT in COVID-19 patients in another study [27].

Despite the effect that vitamin D on COVID-19 patients LFTs is in exploration, many studies are already proved the destructive effect of uncontrolled inflammation on organs that could lead to organ failure, especially kidney and liver, which are the headquartered of the most metabolic functions of the body [28, 29]. Vitamin D has been known to play a critical role in the immune system, and its receptor has been expressed in multiple organs and tissues, including the heart, lungs, kidneys and liver, [4, 12, 30]. As much evidence regarding the consumption of vitamin D has shown, this vitamin could decrease the risk of severe ARDS in COVID patients by decreasing the production of Th1 cells, reducing the inflammatory cytokines generation such as IL-6, IL-8, IL-12, and IL-17, TNFα and inhibits gamma interferon (IFN-γ) and IL-2 to reduce cytokine storm syndrome [4, 12]. This anti-inflammatory effect of vitamin D and the importance of inflammation in organ failures could consider as of the main protective pathways of vitamin D on organs, especially hepatic failure [31].

In addition to known antimicrobial and anti-inflammatory effects, vitamin D metabolites also have a direct action on angiotensin-converting enzyme 2 (ACE2), which serves as the cell surface entry receptor for severe acute respiratory syndrome coronavirus 2 (SARS-CoV-2) [4, 21, 32]. Higher levels of ACE2 have been associated with better COVID health outcomes in previous studies as it enhances the expression of ACE2 [4, 21, 28, 32]. In addition, vitamin D can suppress ACE2 expression, so protectively preventing COVID-19 entry into the tissue cells [4]. In other words, based on this mechanism, the vitamin D, in addition to the protective effect on the SARS-CoV-2, by decreasing the mentioned factors could significantly reduce organ failure in patients. Though the exact mechanism of liver damage in COVID-19 is unclear, several hypotheses include direct viral cytotoxicity through ACE-2, immune-mediated damage, drug-induced liver injury, and passive congestion, have been proposed [12, 28].

Although the current study showed that vitamin D supplementation increased the median serum vitamin D and could affect ALT levels in hospitalized patients with COVID-19, the decision tree analysis indicated that the effect of vitamin D supplementation was not strong enough to cause a node in the tree for none of the liver function tests. A reason for this finding might be that the effects of vitamin D supplementation were lower than other study parameters that are mainly related to disease severity and multiorgan dysfunction, including patient temperature and serum magnesium at baseline; and male gender. Hypermagnesemia, which is defined as serum magnesium above 2.3 mg/dL, is a rare condition in clinical settings but is seen in hospitalized patients with COVID-19. Hypermagnesemia was previously reported to be associated with increased severity and complications in COVID-19 patients; however, the mechanism for this association has not yet been identified but it is hypothesized that the reason for hypermagnesemia might be renal insufficiency, increased magnesium shift from tissues to plasma due to sepsis or microvascular thrombosis [33]. These findings indicate the association of hypermagnesemia with severe COVID-19 [34]. Considering the reported prevalence of hypermagnesemia in ICU admitted COVID-19 patients, it can be hypothesized that the effect of magnesium status on disease severity might have been stronger than vitamin D. Based on the findings of the current study hypermagnesemia was an indicator of no change in ALT which was in line with the findings of the previous study [33]. Furthermore, the association between high serum GGT and ALT and reduced probability of ALT improvement might be an indicator of severe hepatic involvement and implement multiorgan dysfunction in COVID-19 patients. These findings should be evaluated in further studies as the level of ALT in majority of the COVID-19 patients in the current study was within the normal range. Similar findings were observed in terms of AST, which indicate low probability of reduction in baseline AST levels in the presence of elevated serum GGT. However, AST was found to increase after one month in cases with low serum PLT and phosphorus, while elevated serum phosphorus was associated with improved AST levels. Previous studies have indicated that hypophosphatemia was associated with increased mortality and complications in COVID-19 patients [35, 36]. Although the cut-off for phosphorus in the current study was above the cut-off for hypophosphatemia, the findings of this study showed an inverse relationship between baseline serum phosphate level and AST levels on month after the initiation of the disease. Overall, low serum phosphate and increased PLT, as an acute phase reactant, both indicate the severity of COVID-19. Thus, presence of these conditions prone COVID-19 patients to have increased levels of AST after one moth from the disease. Furthermore, decision tree analysis indicated that serum ALP reduced one month after treatment in patients with acidosis, increased neutrophil count and those who underwent ventilation. These findings also indicate the severity of COVID-19 and it is predictable to observe corrections in serum ALP after receiving adequate treatment for these conditions [37–39]. On the other hand, lower serum GGT and RDW indicate a better underlying condition and reduce the risk of severe infection in COVID-19 patients; therefore, were found to be associated with reduced serum ALP [37–39].

Despite the strength of this study (population-based study), some limitations should be acknowledged. Our study enrolled mild to moderate and severe symptomatic individuals, which limits the generalizability of the results to asymptomatic cases of COVID-19. Also, both total and direct bilirubin as parameters associated to liver function were not determined in the present study. Another limitation of the current study was inability to document COVID-19 diagnosis based on RT-PCR due to the shortage in diagnostic PCR kits. At the time the study was conducted patients were diagnosed based on clinical, blood gas, and radiological findings based on national guidelines at the time. Due to limitations in conducting the study in the first two peaks of COVID-19 pandemics, including the patient overload and busy laboratories, serial evaluation of liver enzymes with short intervals was not possible. The authors acknowledge that liver enzymes might be affected by various factors in the acute phase of infectious diseases; however, as the patients did not differ in major risk factors for liver dysfunction between groups, it can be estimated that the observed changes in liver enzymes might be related to the disease.

## Conclusion

Totally, we found that high dose vitamin D supplementation (50,000 IU initial dose followed by 10,000 IU/day for a month) were potentially beneficial in improving ALP marker in COVID-19 patients. The current study also demonstrated that GGT, temperature, serum magnesium, and gender as prognostic factors associated with changes in ALT.

## Supplementary Information

**Additional file 1.** Decision tree analysis to identify the predictors for change in AST and ALP enzymes in COVID-19 patients.

## Data Availability

The datasets collected and/or analyzed during the present study are not publicly accessible due to ethical concerns but corresponding author may provide datasets upon reasonable request.
