# Supplementary material for: High-dose vitamin D supplementation is related to an improvement in serum alkaline phosphatase in COVID-19 patients; a randomized double-blinded clinical trial
Source: J Health Popul Nutr. 2023 Jul 25;42:71. doi: 10.1186/s41043-023-00409-y (PMC10369932; doi:10.1186/s41043-023-00409-y)
Supplement: Supplementary file 1 — Additional file 1. Decision tree analysis to identify the predictors for change in AST and ALP enzymes in COVID-19 patients. [file 41043_2023_409_MOESM1_ESM.docx]

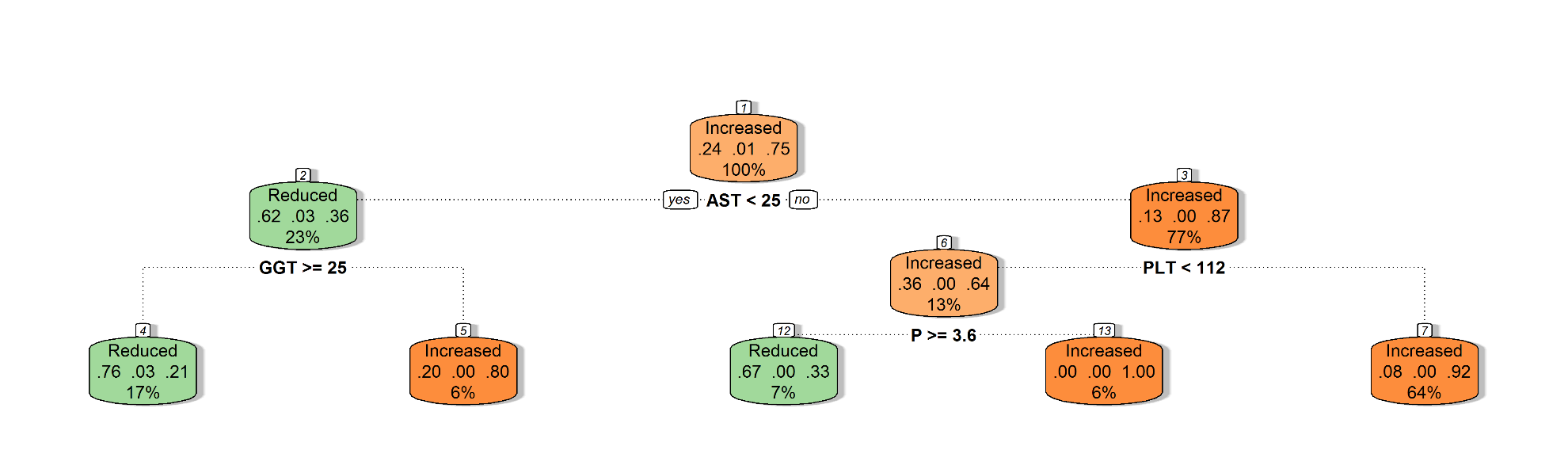


Supplementary file 1.

CART output illustrates a three-level decision tree. The cases partition is based on AST, GGT, PLT, and serum phosphorus as prognostic factors related to changes in AST. The hierarchy consisted of GGT and PLT at level 1, and serum phosphorus at level 2.


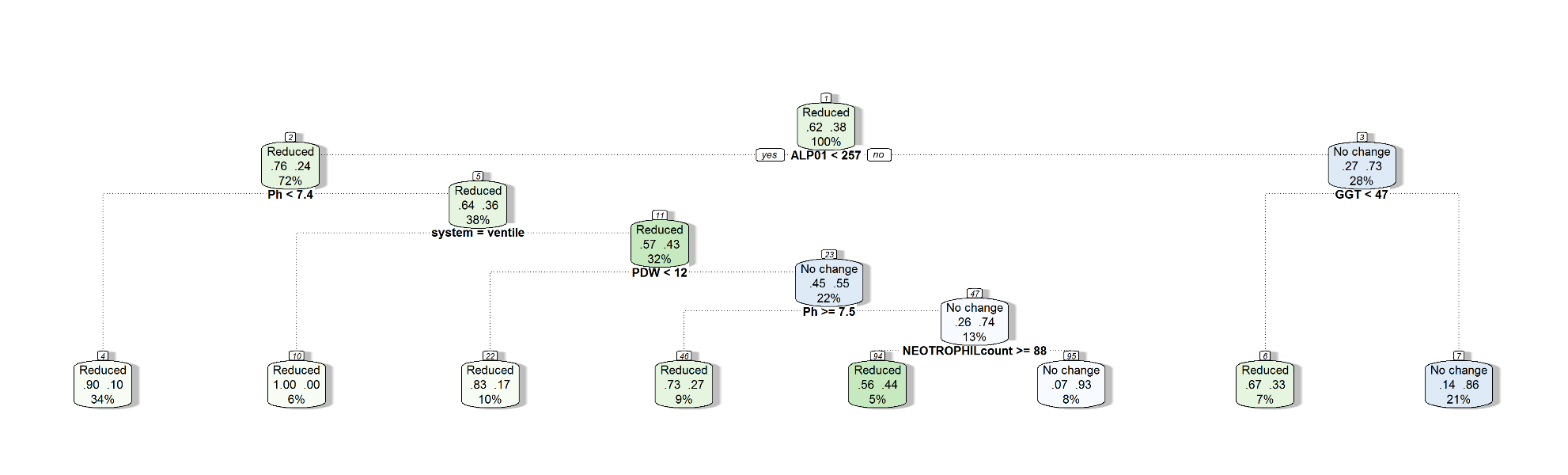


Supplementary file 2

CART output illustrates a five-level decision tree. The cases partition is based on ALP, PH, GGT, ventilation, PDW, and neutrophil count as prognostic factors related to changes in ALP. The hierarchy consisted of GGT and PH at level 1, ventilation at level 2, PDW at level 3, PH at level 4 and neutrophil count at level 5.
